# Tumor Suppressor miRNA in Cancer Cells and the Tumor Microenvironment: Mechanism of Deregulation and Clinical Implications

**DOI:** 10.3389/fonc.2021.708765

**Published:** 2021-10-15

**Authors:** Khalid Otmani, Philippe Lewalle

**Affiliations:** Experimental Hematology Laboratory, Jules Bordet Institute, Université libre de Bruxelles, Brussels, Belgium

**Keywords:** cancer, microRNA, tumor suppressor miRNA, tumor microenvironment, functional regulation

## Abstract

MicroRNAs (miRNAs) are noncoding RNAs that have been identified as important posttranscriptional regulators of gene expression. miRNAs production is controlled at multiple levels, including transcriptional and posttranscriptional regulation. Extensive profiling studies have shown that the regulation of mature miRNAs expression plays a causal role in cancer development and progression. miRNAs have been identified to act as tumor suppressors (TS) or as oncogenes based on their modulating effect on the expression of their target genes. Upregulation of oncogenic miRNAs blocks TS genes and leads to tumor formation. In contrast, downregulation of miRNAs with TS function increases the translation of oncogenes. Several miRNAs exhibiting TS properties have been studied. In this review we focus on recent studies on the role of TS miRNAs in cancer cells and the tumor microenvironment (TME). Furthermore, we discuss how TS miRNA impacts the aggressiveness of cancer cells, with focus of the mechanism that regulate its expression. The study of the mechanisms of miRNA regulation in cancer cells and the TME may paved the way to understand its critical role in the development and progression of cancer and is likely to have important clinical implications in a near future. Finally, the potential roles of miRNAs as specific biomarkers for the diagnosis and the prognosis of cancer and the replacement of tumor suppressive miRNAs using miRNA mimics could be promising approaches for cancer therapy.

## Introduction

MicroRNAs (miRNAs) are small noncoding RNAs that regulate gene expression posttranscriptionally. These RNAs regulate the expression of their target genes by degrading mRNA transcripts or by inhibiting mRNA translation. Dysregulation of miRNA expression is closely associated with cancer initiation, progression, and metastasis. In particular, miRNA gene expression control is critical for the cellular response to environmental stresses, such as starvation, hypoxia, oxidative stress, and DNA damage, largely implicated in cancer. Based on their inhibition of a large variety of tumor-suppressive and oncogenic mRNAs, some upregulated miRNAs act as tumor-promoting oncomiRs undergoing gain of function in cancer development, and other downregulated miRNAs act as tumor suppressors (TS) undergoing loss of function in tumor growth. Numerous miRNAs have tumor suppressive roles in cancer, and their aberrant underexpression leads to abnormalities in cellular processes, such as an increase in apoptosis, enhanced cell growth, invasion and metastasis and decreased sensitivity to treatment through negative suppression of oncogene function.

Studies have shown that a single miRNA complex may bind to more than 200 target genes, which may have a variety of functions, such as transcription factors, receptors and transporters; therefore, it is very challenging to identify target transcripts and pathways that are regulated by specific miRNAs (1). Since microRNAs can target multiple genes, it is not surprising that they impact a variety of cellular pathways. Moreover, numerous miRNAs have dual effects depending on the tumor type. In some cancers, they act as tumor suppressors, while in other cancers, they behave as tumor activators. A global decrease in the expression of mature miRNA in cancer cells causally contributes to the pathogenesis of various cancers. A profiling study of 217 mammalian miRNAs from normal and human cancer samples showed that miRNA expression is globally suppressed in tumor cells compared to normal cells (2). Nevertheless, more specific alterations of individual miRNA expression are also apparent in tumors (3).

An important aspect of the evolution and progression of cancer is the crosstalk between cancer cells and the surrounding microenvironment. This communication has been shown to be mediated by microRNAs. The deregulation in the expression of TS miRNA is not confined to the cancer cell but extends to the tumor microenvironment (TME). The increase in TS miRNA levels in cancer-associated fibroblasts (CAFs) in the TME severely impairs tumor-supporting capacity by reducing proliferation and migration and impairing tumor expansion (4). In addition, downregulation of mature TS miRNA expression in cancer cells and the TME is highly regulated at multiple levels, including transcriptional and posttranscriptional regulation. A variety of posttranscriptional regulation occurs through the biogenesis machinery of miRNA, affecting the components of the miRNA processing machinery such as Dicer1 (5, 6).

In this review, we will first briefly summarize oncogenic network target by TS microRNAs in cancer cells and some of the recent advance in understanding the complex interplay between TS miRNA downregulation and the TME. Given that microRNAs are frequently dysregulated in cancer, an important question is how microRNAs are regulated. We will therefore mainly focus on important mechanisms of the regulation of TS miRNAs and their impaired biological functions in the initiation and progression of various cancers.

## TS miRNAs Downregulated in Cancer Cells and Their Targets

TS miRNAs prevent cancer initiation through the modulation of oncoproteins coding gene expression (7). Many studies have shown that downregulation of some miRNAs is correlated with cancer progression. However, only some TS miRNAs that are often downregulated in cancer cells induce a robust phenotype. This has been demonstrated in various miRNA families that have received substantial attention, including the known tumoral suppressors let-7, miR-15/16, miR-34, and miR-200. The tumor suppressive role of these miRNAs is demonstrated by the repression of their target oncogenic mRNA network, leading to an inhibition of tumorigenesis.

### TS miRNA Promote Apoptosis in Cancer

The miR-15/16 clusters function as TS miRNAs that is often deleted or downregulated in CLL (8). miR-15/16 are reported to be downregulated in various solid tumors, such as melanoma, bladder cancer, colorectal cancer, pituitary adenomas, and prostate carcinoma (9). The main target of miR-15/16 that has been identified is bcL2 (10), miR-15/16 triggers apoptosis by suppressing the expression of bcL2, but it also targets other oncogenes, such as cyclin D1, MCL1, CDC2, ETS1 and JUN, that are involved in cancer progression (9). More recently, ROR1 was discovered as a target of miR-15/16, and it was shown that when the level of ROR1 is lower, miR-15/16 expression is higher (11). ROR1 encodes an oncoembryonic surface protein expressed on the CLL cells of over 90% of patients, and ROR1 is a receptor for Wnt5a that promotes leukemia cell proliferation and survival (11). A causative relationship between p53 loss and a decrease in miR-15/16 in the development of CLL was demonstrated in a mouse model, suggesting that the p53-miR15/miR-16-MCL1 axis may contribute to aggressiveness and drug resistance in CLL cells (9). More recently some TS miRNA were reported to promote apoptosis by targeting Bcl2. miR-140 was shown to inhibits colorectal cancer progression and its liver metastasis by targeting BCL9 and BCL2 (12). Many studies described the regulation of Bcl-2 by miR-148a in various cancer. Recently it has been shown that the miR-148a targets Bcl-2 in patients with Non-Small Cell Lung Cancer (13). Several studies reported that miR-340 exert multiple significant effects in cancer cell by triggering apoptosis. miR-340 decreased the expression of Notch and Bcl2 and increased the expression the levels of BIM and Bax inducing cell apoptosis (14). In CRC miR-340 was shown to trigger apoptosis by targeting RLIP76 (15) and REV3L (16). Moreover, miR-340 mediated apoptosis in SGC-7901 cells (17), by increasing the level of apoptosis-related factors pro-caspase 3, cleaved-caspase 3, and Bax, but inhibited Bcl-2. In ovarian cancer (OC) miR-340 was reported to promote apoptosis by downregulation of NF-κB1 (18). miR-34a was recently shown to increase apoptosis by targeting SYT1 in human colon cancer (CC) (19).

### TS miRNAs Inhibit the Endothelial to Mesenchymal Transition

The EMT mechanism is important for cancer progression, invasion and metastasis. The miR-200 family is an important TS miRNA family that is downregulated in various cancer types and plays an crucial role in the regulation of EMT mechanism (20). The EMT mechanism is regulated by a complex network, including many transcription factors, such SNAIl, TWIST and ZEB (20, 21). In this context, miR-200 inhibits EMT by directly inhibiting zinc-finger E-box-binding homeobox 1 (ZEB1) and SIP1, also known as ZEB2 (22). In addition, ZEB1 exerts negative feedback to regulate the expression of miR-200 by binding to its promoter, inducing reduced miR-200 expression (23). Several tumor profiling studies demonstrated the importance of the miR-200-ZEB1 axis as important for the control of the EMT process. Recently, it has been shown that the disruption of miR-200/Zeb1 is sufficient to induce an effect on EMT and tumor progression (24). Another factor that plays an important role in the regulation of EMT *via* miR-200 is the transforming growth factor β1 (TGFβ). TGFβ is important for the induction and maintenance of stable mesenchymal cells. TGFβ downregulates miR-200 by inducing reversible DNA methylation of the miR-200 loci (25). Moreover, the loss of let-7 levels in breast carcinoma initiates and maintains the oncostatin O (OSM)-induced EMT genetic program, and HMGA2 acts as a master switch in this event (26). The EMT transcription factor SNAI1 represses transcription of the TS miRNA Let-7 in cancer. SNAI1 bind let-7 family promoters to inhibit miRNA transcription (27).

### TS miRNA Inhibit Cell Proliferation

One of the most important function of TS miRNAs in cancer is the ability to suppress the proliferation of cancer cells. In cancer development it is well known that Wnt/β-catenin pathway drives tumorigenesis and cancer progression (28). Emergence evidence has indicated that some TS miRNAs suppress the Wnt pathways. the increase level of miR-340 that act as TS miRNA inhibit the expression of Wnt and represses cell proliferation, while its inhibition induces proliferation (28). miR-340 inhibits the Wnt/β-catenin pathway by targeting LGR5 or FHL2 as well as the CTNNB1-mediated Notch signaling pathway, repressing cell proliferation. This mechanism occur by modulating the expression of β-catenin. Many miRNAs as the well-studied miR-200 were shown to target β-catenin (29). It was also shown that miR-19 inhibits cell proliferation in gastric cancer by targeting Myocyte enhancer factor 2D (MEF2D) (30). MEF2D is a transcription factor of the MEF2 family. MEF2D inhibition leads to repression of the Wnt pathway (30). Recently it was shown that miR-133a-5p suppresses Gastric cancer (GC) proliferation by targeting TCF (31). This gene belong to a group of transcription factors that play an important role in the Wnt pathways by recruiting the β-catenin to enhance the transcription of targeting genes, including some oncogene such c-Myc (31). A recent work showed that ectopic expression of miR-34b-3p that is downregulated in non-small-cell lung cancer (NSCLC) tumoral tissue and the cell lines A549 and H1299, represses cell proliferation and cell cycle progression and induces apoptosis of lung cancer cell lines A549 and H1299 by targeting CDK4 kinase, which is important for cell cycle G1 phase progression (32). miR-34a was recently shown to target SYT1 in human colon cancer. SYT1 confers proliferation advantages to cancer cells and promotes the survival of cancer stem cells. Therefore low levels of miR-34a in colon cancer tissues promote cell proliferation, migration and invasion (19).

### TS miRNA Inhibit Oncogene Expression

The miR-34 family has been identified as a TS miRNA in various cancers by targeting many mRNA oncogenes (33). Low levels of miR-34 are observed in several cancers, including prostate cancer (34), colorectal cancer (35) breast cancer and lung carcinoma (36). Many studies have demonstrated that the aberrant expression of programmed cell death protein (PDL-1) allows cancer cells to evade the immune system and is associated with tumor aggressiveness (37). miR-34 was found to target PDL-1 in NSCLC cell lines (38) and acute myeloid leukemia (AML) (39). In NSCLC, the TS p53 represses PDL1 expression by miR-34, revealing another mechanism by which tumor immune escape is regulated by the p53/miR-34/PDL-1 axis. Given the wide range of oncogenes that are modulated by miR-34, miR-34 represents a novel potential therapeutic target for cancer treatment. The Let-7 miRNA family also acts as a TS miRNA and is silenced in various cancers. The human Let-7 family includes ten mature isoform sequences that are produced from 13 precursors (40). Loss of Let-7 expression is associated with the progression of several human cancers (41–45). Let-7 negatively targets the expression of a wide range of oncogenes, such as the RAS family, HMGA2 (46) and Myc (47). In addition, Let-7 has been reported to play crucial roles in metastasis by targeting HMGA2 in nasopharyngeal carcinoma (42), in breast cancer (44) and in oral cancer (48).

## Mechanism of TS miRNA Regulation in Cancer

**Figure 1:**
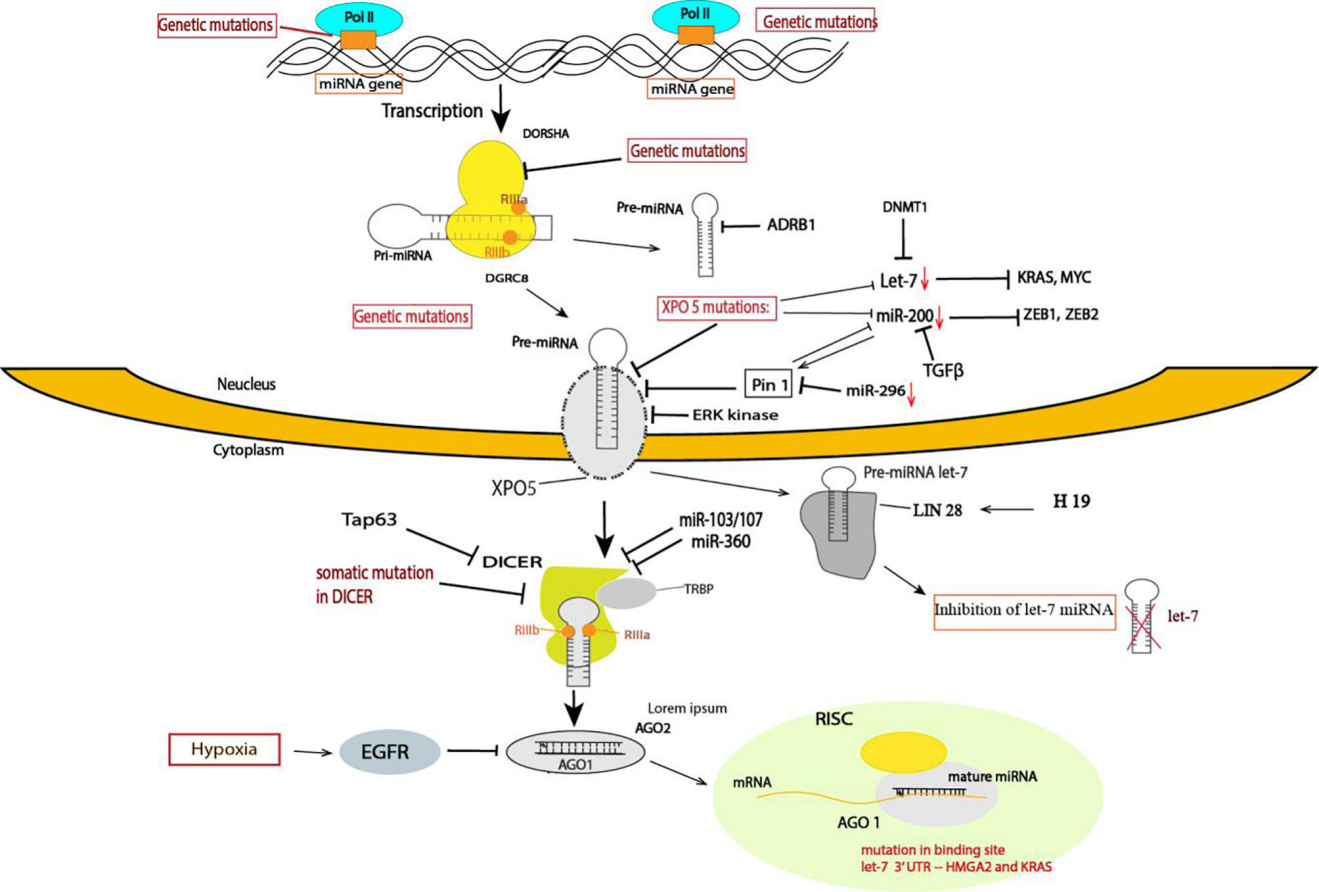
Summary of the mechanism of TS miRNA regulation in cancer. The mechanism controlling TS miRNAs occurs at different steps in cancer, including transcriptional and posttranscriptional regulation. A variety of posttranscriptional regulation occurs in the miRNA biogenesis machinery, which affects the components of miRNA processing. miRNA processing of some TS miRNAs is regulated at multiple levels: at the gene level, through genetic mutations in the miRNA gene and in other components of miRNA processing, such Dorsha, Dicer, and TRBP, and in the binding site of miRNA targets and exportin-5. In XPO mutations, several TS miRNAs are downregulated in cancer, such as miR-200 and let-7. The miRNA transcripts are altered at transcription by several oncogenes, such Myc and TGFB, or by epigenetic modification. LIN 28 proteins specifically block the processing of the pre-miRNA of Let-7. Several studies have highlighted that Dicer downregulation in cancer is caused by several factors, such as somatic mutations, Tap63, miR-360, and miR-103/107. EGFR suppresses the maturation of specific TS miRNAs through phosphorylation of AGO2 and prevents the association of AGO2 with Dicer.

The expression of miRNAs is highly regulated at multiple levels, including transcriptional and posttranscriptional regulation. Posttranscriptional regulation occurs in the miRNA biogenesis machinery, which affects the components of miRNA processing, such Drosha, Dicer, Argonaute 2 and DGCR8 (**Figure 1**).

### Posttranscriptionnal Regulation

#### Downregulation of the miRNA Biogenesis Machinery

The biogenesis of miRNA implies multiple steps. Similar to protein-encoding genes, miRNAs are transcribed by RNA polymerase II from independent miRNA-encoding genes or represent introns of protein-coding genes (49). Initially, miRNAs are transcribed to primary miRNAs (pri-miRNAs), and this transcript is further cleaved by Drosha and DGCR8, generating precursor miRNA (pre-miRNA). Pre-miRNA is exported from the nucleus to the cytoplasm by exportin-5 (XPO5), where it is processed by Dicer into mature miRNA, which forms what is called the miRNA-induced silencing complex (miRISC) containing the Argonaute 2 protein and glycine-tryptophan protein (GW182). The complex is formed where the miRNA and mRNA target interact (50).

Impaired miRNA biogenesis is one of the mechanisms for the global downregulation of miRNAs observed in various cancers. It was shown for the first time in leukemic cells that impaired miRNA maturation contributes to the downregulation of TS miRNAs (51). Another study confirmed that impaired miRNA biogenesis enhances cellular transformation and promotes tumorigenesis. The authors demonstrated that the global repression of miRNA maturation occurs by the expression of short hairpin RNAs (shRNAs) by cancer cells targeting three different components of the miRNA processing machinery, driving a substantial decrease in steady-state miRNA levels and a transformed phenotype (6). In line with this finding, downregulation of Drosha and Dicer, two key component proteins involved in the miRNA processing pathway, was reported in various cancers and is often associated with a poor clinical outcome (41, 42, 51, 52).

Dicer downregulation in cancer cells is induced by several mechanisms. For example, Tap63 is a transcription factor that activates the transcription of Dicer by directly binding to the promoter; low levels of Tap63 induce a downregulation of Dicer (53), and loss of Tap63 has been found to be associated with various cancers. The expression of Dicer can also be repressed by direct targeting of the 3’UTR by miR-103/107 (54) and miR-360 (55).

Nevertheless, Drosha and Dicer1 can also be expressed at higher levels in some cancers, such as in cervical cancer, prostate adenocarcinoma (56), AML (57) and in precursor lesions of lung adenocarcinoma (58). Moreover, a specific impairment of TS miR-15/16 family processing leads to its reduced levels. This defective processing is linked to the RNA-editing gene ADRB1, an RNA-specific deaminase that is known to modify pre-miRNA and interfere with miRNA processing (51). However, the variable expression of those enzymes in human cancer indicates a complex and likely a cancer-specific dependency of the miRNA biogenesis mechanism in cancer development.

In addition to those crucial enzymes, other miRNA processing pathway enzymes, such as AGO2 and TRABP2, have also been reported to be downregulated. Epidermal growth factor receptor (EGFR), which is an oncogene in human cancer, suppresses the maturation of specific TS miRNAs through phosphorylation of Argonaute 2 (AGO2), which prevents the association of AGO2 with Dicer and inhibits miRNA processing from precursor miRNAs to mature miRNAs in response to hypoxic stress (59).

The biogenesis of miRNA is regulated at diverse steps: transcription of miRNA genes, processing by Dicer and Drosha and transportation of pre-miRNAs to the cytoplasm by XPO5. Nuclear export of pre-miRNA by XPO5 is a crucial step in miRNA biogenesis. Recently, posttranscriptional modification of XPO5 or abnormal expression levels of XPO5 proteins leading to decreased mature miRNA production have been found to be associated with tumorigenesis. Importantly, the phosphorylation of the XPO5 protein by ERK kinase, followed by its cis/trans isomerization by prolyl isomerase (Pin), impairs the ability of XPO5 to transport pre-miRNA, inducing a downregulation of mature miRNA expression and leading to increased migration, cell proliferation and invasion in hepatocellular carcinoma (HCC) (60). A recent study showed that Pin1 downregulates the expression of TS miRNAs by modulating the phosphorylated serine-proline (pS-P) motif of the XPO5 protein (61). Downregulation of miRNA in cancer has a significantly negative correlation with Pin1 expression. In prostate cancer, miR-296 functions as a TS miRNA and induces an inhibition of prostate cancer cell proliferation by targeting Pin (62). It has also been shown recently that targeting Pin1 exerts potent antitumor activity by inhibiting tumor metastasis in pancreatic ductal carcinoma (63). Downregulation of the miR-200 family contributes to Pin1 overexpression and promotes tumorigenesis in breast cancer (55, 56). It was demonstrated that Pin1 inhibits the expression of miR-200b, which can act as a positive feedback loop of Pin1 overexpression in cancer (61).

#### Inhibition of TS miRNA by Long Non Coding RNAs

LncRNAs are a type of a non-coding RNA which is longer than nucleotide in length (64). LncRNAs binds with miRNAs as a competitive endogenous RNA, which preventing miRNAs from binding to mRNA targets and consequently antagonized their functions (65, 66). LncRNAs was shown to play a crucial role in in multiple biological function, including apoptosis, cell proliferation and differentiation. Recently, lncRNAs have been reported as an important regulatory factors in tumorogeneisis (67–70). LncRNAs was shown in many cancers type to be enriched and target negatively the expression of TS miRNAs. In breast cancer steam cell (BCSCs) and breast tumor samples, lncRNA H19 was shown to be expressed in high level (45). The ectopic expression promotes breast cancer development. Conversely, silencing of lncRNA H19 represses the BCSC progression. Mechanistically, lncRNA H19 act as a molecular sponge to regulate the expression of miRNA Let-7. H19 increases the expression of LIN28 and in its turn bind the conserved terminal loop of precurssor miRNA-let-7 elements preventing DICER to cleave pre-Let-7 transcript, inhibiting the production of mature Let-7 miRNAs (45). LncRNA LINC01018 inhibits the progression of AML by targeting miR-499-5p to regulate PDCD4 (71). Moreover, lncRNA SNHG15 was reported to executed oncogenic proprieties in GC by impairing miR-506-5p expression (72). A very recent study has shown that LncRNA LINC01410 induced by Myc accelerates Glioma progression *via* decreasing the expression of miR-506-3p and Modulating Notch2 Expression to motivate Notch signaling pathway. It was also shown that LINC01410 promotes the progression of cholangiocarcinoma through modulating miR-124-3p/SMAD5 axis (73). Moreover, lncRNA metastasis-associated lung adenocarcinoma transcript 1 (MALAT1) has been shown to be overexpressed in MM and directly target the expression of miR-188-5p that serves as TS miRNA (74).

### Transcriptional Regulation

#### Downregulation of TS miRNA by Aberrant Transcriptional Control in Cancer

Considering that approximately half of the miRNA genes are usually found in the introns of coding genes or long noncoding genes, while the other miRNAs are derived from their own promoters (75), alteration of transcription factor in cancer cells induce a downregulation of TS miRNA in cancer cells.

##### Oncogenic Transcription Factors Repression

The transcription of TS miRNAs is often repressed by oncogenic transcription factors. Myc is an oncogenic transcription factor that regulates the expression of various miRNAs. However, the main function of Myc is the repression of a wide repertoire of miRNAs that have a tumor suppressive characteristic, many of which have anti-proliferative and pro-apoptotic effects, such as miR-34, Let-7 and miR-15a/16-1 (76). Myc can also repress the expression of TS miRNA indirectly by activating the expression of LIN28A and LIN28B, which are required for repression of the Let-7 miRNA (77). Many studies have demonstrated a role of miRNAs in other transcription factor networks. For example, the miR-200 family regulates the expression of ZEB1 and ZEB2, which in turn represses the transcription of miR-200 genes, generating a complex feedback loop, and strongly regulates epithelial differentiation in many cancer types (22, 23).

Several studies has investigated the role of estrogen and its nuclear receptor (ER) in modulating miRNA expression in cancer especially in breast cancer cells. Estrogen is involved in various pathological processes through ER-mediated transcriptional gene regulation. One of the estrogen-regulated miRNAs, miR-34b, has been functionally identified and validated as a TS miRNA downregulated by estrogen (78). Estrogen repress the expression of miR-34b through the interaction of ER and p53 which serves as transcriptional factor of miR-34b (78). Another study has shown the identification of differential expression profile of miRNAs in breast cancer cell line MCF-7, in response to oestrogen treatment, miR-107, miR-570 and miR-618 were shown to be strongly downregulated (79). A very recent study demonstrated that miR-1291 targets the expression of ER and exert an antitumor effect. miR-1291 inhibits the proliferation capacity and metabolism status of pancreatic cancer cell line PANC-1 and the breast cancer cell line MDA-MB-231 by targeting the ER. miR-1921 form a regulatory axis with ER that controls the proliferation and cell metabolism by targeting the expression of Carnitine palmitoyltransferase 1C (CPT1C) in breast and pancreatic cancer (80). Moreover, miR-26a and miR-26b were identified as novel target of ER in breast cancer. ER stimulate Myc to suppress the expression of miR-26a/b mediating cell proliferation by targeting CHD1, GREB1 and KPNA2 (81).

The oncogenic protein Yes-associated protein (YAP) that is upregulated in cancer is reported to mediate the global miRNA suppression in tumors by regulating miRNA processing. YAP exerts its function through binding and sequestering DDX17 (DEADBox Helicase 17, also known as p72) away from Drosha and DGCR8 in a cell-density-dependent manner, reducing the processing of pri-miRNA to pre-miRNA by Microprocessor, thus limiting overall miRNA production. At higher cell density, YAP is inactivated by exclusion from the cell nucleus, thereby allowing p72 to associate with microprocessor and pri-miRNAs, resulting in enhanced miRNA biogenesis (82). The activation of YAP causes widespread miRNA suppression in cells and tumors resulting in the posttranscriptional induction of oncoproteins such Myc, suggesting an important role of miRNA biogenesis in YAP-induced tumorigenesis. Myc was shown to be responsible of suppression of miRNA biogenesis in many cancer type. However, the authors considered that suppression of a certain subset of miRNAs by YAP might lead to posttranscriptional enhancement of target genes important for tumorigenesis and growth (82). YAP was shown to be a target of some TS miRNAs in cancer such miR-591 and miR-200 BC (83, 84) and in HCC (85).

##### Tumor Suppressive Transcription Factors Loss

Downregulation of TS miRNA can also result from loss of TS transcription factors. However, transcription of miRNAs with TS effects is often activated by transcription factors that have their own important growth suppressive effects. p53 is a transcription factor with a TS function that plays a pivotal role in the regulation of apoptosis and cell cycle progression and governs the cellular response to DNA damage. Many TS miRNAs are under transcriptional control of p53, such as the miR-34 and miR-200 families. The miR-34 family, which is commonly deleted in human cancer, has been shown to be an important component of the p53 TS network (86, 87). The global expression analysis of miRNA levels after genotoxic stress that induces the upregulation of p53 has identified that miR-34 family members a, b, and c are strongly upregulated miRNAs (86). Similar to the known target set of miR-34 family-regulated genes, p53-responsive genes are highly implicated in apoptosis and cell cycle progression. Given the function of the wide range of target genes that are modulated by miR-34, it is clear that the p53 network suppresses tumor formation and is coordinated by transcriptional activation of miR-34 (83). Several other TS miRNAs are regulated by p53 miR-15/16, miR-107 and miR-145. Recently, a novel axis involving p53, miR-30a and ZEB2 was identified that controls tumor progression and metastasis in triple-negative breast cancer (88). The miR-30 member family was shown to act as a TS miRNA in breast cancer growth and metastasis (89). In particular, the expression of miR-30a (both miR-30a-5p and miR-30a-3p) was significantly altered in human breast cancer carrying the p53 gene alteration (88). Additionally, p53 regulates the expression of the component of miRNA biogenesis, either directly by binding to Drosha or indirectly (90). p53 interacts with Drosha through the association with DEAD-box- RNA helicase p68, thus activating the processing of pri-miRNA generating the precursor miRNA. Also the inactivation of transcriptionally p53 induces the decrease in the miRNa processing activity through the inhibition of the functional assembly between Drosha and p68 (90). The p53 family member p63 has been shown to activate the transcription of Dicer1 and miR-130b (53). Inactivated p63 by association with the mutant-p53 induces a downregulation of Dicer1 expression, resulting in low levels of mature miRNAs and increased metastasis (53).

Many tumor suppressors are mutated or repressed in cancer, such as PTEN and p53, resulting in loss of expression of miRNAs with antitumorigenic effects (91, 92). The function of p53 is deregulated in many cancer types, and it’s mutated in more than 50% of human cancers (93), resulting in an oncogenic functions known as a gain-of-function (GOF) activities promoting tumorigenesis (94, 95). At molecular level, GOF acquired by mutant-p53 forms show a dominant-negative (DN) effect either through the binding, sequestration, and the inactivation of tumor suppressor proteins (96), or through the binding to the gene promotor and enhancing its transcriptional regulation (97). However, mutant-p53 was described as transcriptional factor of some miRNAs (97). The widespread nature of mutant-p53 in cancer has suggested the relationship between mutant-p53 GOF activities and the deregulation of miRNA biogenesis observed in cancer (98). Mutant-p53 was shown to suppress the expression of miR-26a by disrupting p68-Drosha complex assembly (99). Another important work demonstrated that mutant-p53 proteins downregulates the expression of miRNAs not only at transcriptional but also at post-transcriptional level by direct binding and sequestering RNA helicases p72/82 through its N-terminal domain from microprosessor complex, hindering the association with Drosha/p72/82 and pri-miRNA and leading to the inhibition of the biogenesis of a subset of miRNA that positively regulated by p72, promoting cell survival and cell migration (100). Recently, in colorectal cancer (CRC) cell line SW480/OxR with mutant-p53, LINC00460-miR-149-5p/miR-150-5p-mutant p53 feedback loop was found to play an important role for oxaliplatin resistance in CRC. In this study the authors demonstrated that the upregulation of LINC00460 in CRC cell line promotes mutant-p53 expression through competitive binding of miR-149-5p/miR150-5p, and in turn mutant-p53 induces the expression of LINC00460, thus forming a positive feedback loop that drives oxaliplatin resistance in SW480/OxR cells (101).

#### CpG Methylation and Histone Modification Deregulated TS miRNAs in Cancer

MicroRNA-mediated gene silencing is considered one of the major epigenetic modifications that change the genomic structure and thereby influences gene expression without modifying the DNA sequence (102, 103). Increasing evidence supports the idea that miRNAs can also be deregulated in cancer by abnormal CpG methylation or histone modification. A signature of miRNAs hypermethylated in metastatic cell lines has been found in colon, melanoma, and head and neck cancers. miRNA-34, miR-148a and miR-9 were found to be associated with CpG island hypermethylation in metastatic cell lines in primary colon, oral, breast, lung, gastric head, neck, melanoma and pancreatic cancers (104, 105). miR-200 has been found to be downregulated in the stem-like cell fraction isolated from metastatic breast cancer, and it has been demonstrated that the miR-200c-141 cluster is silenced by CpG hypermethylation, whereas the miR-200b-200a-429 cluster is repressed through polycomb group-mediated histone modification (106). Recently, EZH2 a histone methyltransferase was shown to be responsible for the epigenetic regulation of TS miRNAs in HCC. EZH2 inhibit the expression of miR-200c promoting the hepatocarcinogenesis through increasing the expression of BMI1 (107). More recently, miR-22 that act as TS miRNA in wide variety of cancer such as colorectal cancer, gastric cancer, breast cancer, hepatocellular carcinoma and lung cancer, was shown to be subject to epigenetic regulation by methyl-CpG-binding protein 2 (MeCP2) in gastric cancer (108). MeCP2 silenced miR-22 by binding to their upstream element, including not only the promotor but also the distal enhancer (108). Several TS miRNAs have been found to be inactivated by hypermethylation in multiple myeloma, such as miR-188-5p (74), miR-34b/c and miR-203 (109). Very recently, it have been shown that the SWitch/Sucrose Non-Fermentable (SWI/SNF) complex, a member of the family of ATP-dependent chromatin remodeling complexes is involved in miRNAs expression regulation, SMARCA4-SWI/SNF complex binds to the miR-222 enhancer, increasing the expression of miR-222, who acts as TS miRna (110). SWI/SNF complex plays a relevant role in pathogenesis. SWI/SNF is one of the most frequently mutated epigenetic regulators in cancer (111).

#### Inhibition of TS miRNA Transcription by Genomic Deletion

miRNA genes throughout the genome are usually not disturbed, and SNPs in miRNA genes are significantly lower in comparison to the whole genome (112). Nevertheless, miRNA coding sequences are often amplified or deleted in cancer, similar to TS genes and protein coding oncogenes (113). Genomic alterations are critical in oncogenesis, and an increasing number of single nucleotide polymorphisms (SNPs), mutations and chromosomal translocations have been shown to be capable of driving cancer (114). In addition, genomic alteration of miRNA may be an important mechanism responsible for miRNA deregulation reported in several tumor types (115). A very recent work characterized and identified several significant mutations in miRNA gene sequences, and most of these mutations were typically observed in TS miRNAs (112). This finding coincides with previous work suggesting the pattern of loss-of-function mutations observed in suppressor genes (116). Moreover, a somatic mutation in Dicer promotes cell proliferation and regulates the expression of differentiation genes partially through silencing the TS miRNA let-7 family (117).

Moreover, it was shown that mutations in human Dicer are recurrent in several cancers (118, 119),, suggested by partial loss of function of the miRNA processing machinery in human tumors. Previously, it was shown that the Dicer1 single copy deletion in human cancers provides a relevant mechanism for impaired miRNA biogenesis, suggesting a broad role for Dicer1 as a tumor suppressor. A very recent report highlighted the importance of a Dicer1 mutation in anaplastic sarcomas of the kidney (120), and other recent publications have shown that Müllerian adenosarcoma tumorigenesis is characterized by a somatic Dicer1 mutation (121). A Dicer1 hotspot mutation has been shown to be involved in the dysregulation of hormone synthesis in ovarian Sertoli-Leydig patients (122). Sarcoma and lung cancer exhibit impaired miRNA processing without loss of the wild-type Dicer1 allele. Full deletion of Dicer1 induces inhibition of tumorigenesis (123), indicating that cancer cells need a certain minimum level of Dicer expression for tumorigenesis or cell survival. The presence of Dicer knockout cell lines, such as embryonic stem cells, indicates that this phenomenon may be cell type-specific (124).

Mutations in other components of the miRNA processing machinery occur, as in the TARBP2 miRNA processing component in colon cancer and in human ovarian cancer cell lines. Mutations have also been described for Dicer1 and Drosha, which show no correlation with those genes expression levels. However, the low expression of Dicer1 and Drosha is associated with a poor outcome in patients with ovarian cancer (125). A very recent published work demonstrated that a somatic Dicer1 RNase llla/b mutation hotspot previously studied in endometrial cancer (119) impedes 5’ pre-miRNA biogenesis and activity, implying significant changes in targets in a few specific miRNA families, including let-7 and miR-15/16. These Dicer1 RNase llla/b biallelic mutations are notably present in uterine cancer (126).

Disrupting the pairing between miRNA and mRNA targets by mutation is another type of oncogene activating event. Let-7 represses HMGA2, a chromosomal translocation previously associated with tumors, and disrupts the repression of HMGA2 by Let-7 (127).

A mutation in the gene encoding XPO5 induces a decrease in the export of TS miRNA in cancer. Two of the best examples are Let-7 and miR-200, which are downregulated in the XPO5 mutant. Importantly, impairment of TS miRNAs in the XPO5 mutant leads to an increase in their target oncogenes, such Myc and K-RAS for Let-7 and ZEB1 for miR-200 (128). A small but significant mutation in miRNA genes or in their biogenesis machinery can alter miRNA processing, which ultimately influences the level and function of mature miRNAs. This highlights the interplay between genetic variation in miRNA synthesis and posttranscriptional regulation of miRNA biogenesis.

## TS miRNAs in Tumor Microenvironment

### TS miRNAs Modulate the Tumor Microenvironment

**Figure 2:**
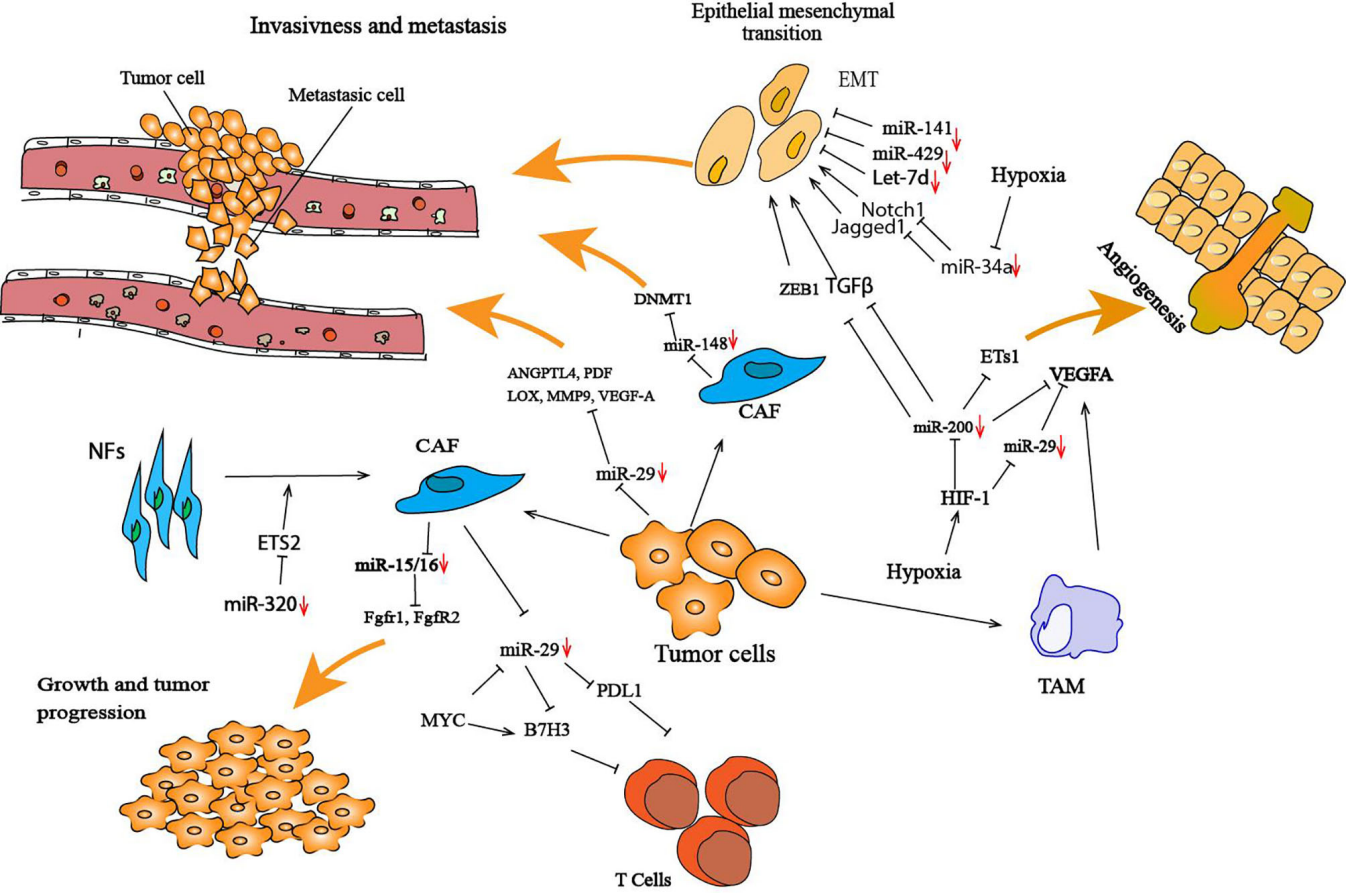
Illustration of cancer cells and the tumor microenvironment-deregulated TS miRNAs that lead to tumor development and progression. In the tumor microenvironment, miRNAs regulate the interaction between cancer cells and their surrounding microenvironment by targeting several genes involved in this interaction. Several TS miRNAs are deregulated in the tumor microenvironment; for example, miR-29 and mR-148 are both downregulated in CAFs, and this deregulation promotes cancer cell invasion and metastasis. These miRNAs have been found to negatively target several genes that are crucial in enhancing cancer metastasis, such DNMT1 for miR-148 and ANGPTL4, PDF, LOX, MMP9, VEGF-A for miR-29. Hypoxia in the tumor microenvironment has a protumorigenic role by altering the expression of TS miRNAs, such as miR-200, miR-34, miR-29 and miR-15/16. Inhibition of miR-200 can significantly increase the expression of Ets-1, resulting in the promotion of angiogenesis. HIF-1 is one of the most studied genes in hypoxia that plays a crucial role in the tumor microenvironment and has been associated with the downregulation of miRNAs. CAFs play an important role in cancer cells during initial cancer and metastasis processes. miR-320 is a TS miRNA that is known to play a part in transforming NFs to CAFs. miR-320 targets ETS2, a cancer-specific transcription factor, resulting in increased oncogenic secretome secretion, and this oncogenic secretome converts NFs to CAFs in the tumor microenvironment (97).

The dynamic crosstalk between cancer cells and normal cells in the TME by the secretion of molecules such as cytokines, chemokines and growth factors has been largely studied, but other molecules, such miRNAs, are also involved in the regulation of several physiological intercellular dialogues and play a significant role in disease progression, including cancer. The TME is very heterogeneous and incorporates various cell types, such as immune cells, fibroblasts, and endothelial cells, and the extracellular matrix (ECM). miRNA can affect the tumor immune microenvirement by regulating the function of immune cells, which in turn modulates the progression of tumor cells (129). TS miRNAs show the ability to act on both stromal and tumor cells to inhibit cancer. Recently, in prostate cancer, it has been shown that the TS miR-15/16 is underexpressed in fibroblasts surrounding prostate tumors. Downregulation of those miRNAs in CAFs promotes the progression and growth of tumors through a reduction in Fgf-2 and repression of its receptor Fgfr1. Fgf-2 and its receptor Fgfr1 play a crucial role in both stromal and cancer cells in enhancing cancer cell survival (4). This suggests a molecular pathway in which both miR-15/16 and its target cooperate to promote tumor progression and invasiveness through concurrent activity on cancer and stromal cells. It has been recently shown that downregulation of exosomal miR-148b from CAFs promotes endometrial cancer cell invasion and metastasis. miR-148b acts as a TS by directly suppressing the expression of DNMT1, which plays a crucial role in enhancing cancer metastasis by inducing EMT (130) (**Figure 2**).

miRNA-29b, a TS miRNA, is downregulated in various types of cancer, including lymphoma, nasopharyngeal carcinoma, glioblastoma and osteosarcoma (87, 88). miRNA-29 possesses antitumoral effects, including inhibition of proliferation and migration. Moreover, miR-29b has been shown to alter the TME by affecting angiogenesis and collagen remodeling to inhibit metastasis and to induce antitumor epigenetic modulation. miR-29b forms a regulatory axis with GATA3 and acts as a TS by modulating the TME in breast cancer. Low expression of miR-29b increases metastasis and promotes a mesenchymal phenotype (127). Mechanistically, miR-29b inhibits metastasis by targeting various genes involved in angiogenesis and collagen remodeling, including ANGPTL4, platelet-derived growth factor (PDF), LOX, matrix metalloproteinase 9 (MMP9) and vascular endothelial growth factor-A (VEGF-A). miR-29 has been shown to directly inhibit the expression of B7-H3 (131). B7H3, known as an inhibitory immune checkpoint ligand (132), is highly expressed on the vascularization surrounding breast, lung and kidney tumors (133) and is responsible for the suppression of T cell function in several cancers (134). A recent study showed a triangular regulation between Myc-miR-29 and B7-H3 in medulloblastoma (MB) tumoral cells, in which Myc directly regulates the expression of B7-H3 and indirectly regulates the expression of miR-29. The upregulation of B7-H3 by Myc promotes MB angiogenesis and can be inhibited by miR-29 overexpression *via* miR-29-mediated B7-H3 downregulation (131) (**Figure 2**).

More important, TS miRNA have shown abilities to enhance immune response, by diminishing immune-suppressive mechanisms and or suppress the STAT3 pathway. Cancer cells may also downregulate some TS miRNA that target negatively the expression of immunosuppressive factors and inhibitory costimulatory molecules involved in the inhibition of effector T-cell activation. For example miR-322 that behaves as TS miRNA, suppress the expression of galectin-3 (135), while miR-181 block the biosynthesis of TGF-β receptor 1 and TGF-β receptor associated protein 1 (TGFBRAP1) suggesting that miR-181a may enhance immune response by suppressing the immunosuppressive TGF-β pathway (136).

### Factor Influencing TS miRNA Expression in the Tumor Microenvironment

Some circulating and intracellular miRNAs are dysregulated in cancer, and many studies have shown that the alteration of miRNAs in cancer cells modulates the TME. The alteration of the expression of miRNA profiles is induced not only by dysregulation in signaling pathways in different cancer types but also by general events such as hypoxia (137). Some studies have shown that miRNAs are associated with some signaling pathways in response to hypoxia and play an important role in hypoxic stress. miR-200 has been shown to be downregulated by hypoxia and hypoxia-inducible factor-1 (HIF-1), and this inhibition of expression induced high Ets-1 expression that promotes angiogenesis (138). HIF-1 is one of the most reported genes in hypoxia, which plays an important role in the TME and is associated with miRNA downregulation (137).

Another regulatory mechanism is when the TME alters not only the expression of miRNAs but also negatively modulates their function. Hypoxia attenuates VEGF-A repression by inducing the cytoplasmic translocation of the nuclear ribonucleoprotein L (hnRNP L), which binds the VEGF-A 3’UTR in CA-rich elements, thereby inhibiting miRNA silencing activity (139). The expression of VEGF-A by tumor-associated macrophages (TAMs) plays an important role in metastasis and tumor progression (139). This gene is targeted by several TS miRNAs, such miR-15/16 and miR-29, and downregulation of this miRNA by hypoxia induces an increase in VEGF-A that promotes angiogenesis and initiates tumor vascularization (140). Moreover, hypoxia increase the expression of EGFR, which impairs TS miRNA maturation (59). Another new mechanism was identified in which tumor hypoxia decreases TS miRNAs by inducing a downregulation of Dicer expression. In such events, hypoxia regulates the expression of Dicer by epigenetic mechanisms through inhibition of the oxygen-dependent trimethylated histone H3 lysine (H3K27me3) demethylases KDM6A/B (141). This downregulation of Dicer induces a decrease in miR-200 family processing, leading to an increased level of ZEB1 and activation of the EMT in breast cancer.

## Diagnostic and Prognostic Value of TS miRNA in Cancer

Several studies have shown that the quantification of some circulating miRNA levels in peripheral blood can be used to detect tumors and to predict progression, prognosis and response in many cancer types (2). miRNAs are more stable than mRNAs and exhibit tissue specificity, making them potential diagnostic and prognostic biomarkers. Downregulation of circulating TS miRNA is implicated in the progression of many cancers and is associated with a poor clinical outcome. Circulating miRNAs in human plasma or serum are protected from endogenous RNA activity by residing in macrovesicles, exosomes, or apoptotic bodies. These miRNAs can easily be quantified in human plasma or serum and can distinguish patients with cancer from healthy controls (142). The expression levels of some miRNAs, such as Let-7, miR-10b and miR-155, can distinguish patients with cancer from healthy donors in the majority of patients in a non-cancer-specific manner (143). This finding suggests that an individual cancer can exhibit a specific miRNA profile, which may help in discriminating cancer types. Another study demonstrated that some miRNAs can also detect stage I or stage II breast cancer patients at significant levels compared to healthy donors, making them attractive candidates for early cancer diagnosis (144). Changes in miRNA expression can also correlate with metastasis and can therefore be used as a prediction for distal metastasis. Downregulation of miR-361 has been implicated in the progression of lung cancer (145), breast cancer (146), glioma (147) and papillary thyroid carcinoma. Reduced miRNA-361 expression in tumoral tissue is associated with shorter overall survival in lung cancer (145) and colon cancer (148). The Let-7 miRNA family is repressed in many different types of cancer, including breast cancer (41), lung cancer (46), gastric cancer (43) and nasopharyngeal carcinoma (42). Lower levels of Let-7 are correlated with aggressiveness and a poor prognosis, while increased levels are correlated with a good prognosis and prolonged patient survival (44, 143, 149). Lower expression of the Let-7 family is detected in metastatic sites than in primary tumors in gastric and breast cancer (21, 43, 133). Increased levels of Let-7 in breast cancer are associated with lower liver and lung metastases, while its downregulation increases the number of metastases (41, 109). Recent studies have demonstrated that the miR-200 family can be used as a prognostic marker in various cancers (150, 151). Some miRNA families, due to their heterogeneous expression in different tissues and between different cancers, have variable predictive value for patient outcomes. A very recent meta-analysis showed that the miR-200 family may serve as a prognostic biomarker for cancer. It should be used in different tumor tissues based on different members of the miRNA family and requires specific interpretation (152, 153). Taken together, these data show that miRNAs can be useful specific cancer biomarkers to predict clinical outcome and can help in the discrimination of different cancer subtypes.

## Treating Cancer With TS miRNA

Globally, lower levels of TS miRNAs are causal agents in several cancers and are correlated with tumor progression and poor patient outcome. Owing to the ability of miRNAs to behave as TS, miRNAs have become potential candidates and provide an opportunity for new cancer therapy strategies. Several miRNA-based therapeutics have reached the preclinical stage, and miR-122 reached phase II trials for the treatment of hepatitis (154, 155). miR-34 was the first to undergo phase I clinical trials in oncology for the treatment of several solid and hematological malignancies (156). miRNA-based therapeutics can be divided into miRNA mimics related to the restoration of TS miRNA expression and inhibitors of miRNAs known as antagomiRs. Therapeutically replenishing TS miRNAs using miRNA mimics has been considered for the clinical modulation of miRNAs. Various studies have shown that the restoration of TS miRNA has significant antitumor activities. Several clinical studies using miR-34 mimics encapsulated in lipid nanoparticles have shown their great potential as therapeutic agents, and they are considered the most advanced miRNA therapeutics for cancer. A recent study showed that treatment with miR-34 encapsulated in lipids induced a significant decrease in NSCLC tumors in a mouse model (157). A subsequent study showed that a combination treatment with miR-34a and Let-7 using lipid nanoparticles resulted in a significant decrease in tumors, and combinatorial treatment with the EGFR inhibitor EROLINIB resulted in the strongest synergistic effect in inhibiting the proliferation of NSNLC (158). Another miRNA that has been used in preclinical studies is miR-200. In a lung cancer xenograft model, the treatment of tumors with miR-200c mimic increased cellular radiosensitivity by directly targeting, in addition to the repressor ZEB1, the genes of oxidative stress response sestrin1 (SESN1), NF-related factor 2 (NRF2) and peroxiredoxin 2 (PRDX2), thereby increasing the levels of reactive oxygen species (ROS) (159). Several TS miRNAs delivery systems are under development. miRNAs could emerge as a key technology for the treatment of cancer and other diseases if clinical trials using miRNAs confirm their preliminary encouraging success.

## Discussion and Conclusion

Cancer is an extremely complex disease that is driven by a combination of various aberrantly regulated processes, such as, deregulation of the epigenetic machinery. miRNAs involved in cancer are divided in two groups: the oncomirs that can induce tumor initiation and progression, and their expression are higher in cancer cells. The second group is TS miRNAs, that are downregulated in cancer cells, prevent cancer beginning and progression of cancer through suppressing the expression of various oncogenes. In this review we have highlighted recent advances in the understanding of the role of TS miRNAs, and how TS miRNAs affect the progression of cancer cells and modulate the TME by antagonizing processes that are crucial for cancer cells development. We also emphasized the most important mechanisms of the regulation that specially silence the expression of TS miRNA.

TS miRNA are downregulated in cancer tissues, upon re-expression they suppress various processes relevant to tumorigenesis, including growth and proliferation as well as invasion and metastasis. However, reducing TS miRNA expression in cancer cells, allow cancer progression by reducing apoptosis, inducing cell survival and suppressing tumor invasion and metastasis. In some instances, the overexpression of these miRNAs was shown to be effective in inhibiting cancer proliferation, increasing the sensitivity of cancer cells to apoptosis signals, and suppressing tumor invasion, metastasis and *in vivo* angiogenesis, by targeting the production of oncogene (127, 140).

Accordingly, the restauration of the expression of TS miRNA to inhibit cell proliferation and growth emerges as a new target strategy that could be used in many cancer types, given that TS miRNA play pivotal role in a wider range of human cancers. Importantly, many studies have shown the efficiency of combined treatments with current chemotherapies. Currently, there is emergence of several potential TS miRNAs that have been demonstrated to negatively target cancer cells stability in various pre-clinical cancer models. Further studies are required to evaluate the anti-cancer efficiency of the specific TS miRNA in multiple pre-clinical models and eventually in clinical trials.

Under the pressure of immune system, cancer cells develop multiples mechanism to evade attack by immune system. One of the mechanism used is the deregulation of the expression of miRNA in cancer cell and TME. Tumor cells aberrantly express a number of miRNAs, and this abnormal miRNA expression profile is characterized by the downregulation of TS miRNA and by the maintenance of an increased levels of oncogenic miRNA (oncomiR).

Generally, the expression of miRNA in cancer show a global downregulation (2), this downregulation affect especially TS miRNA maturation. Due to their role in the regulation of gene expression, the expression of mature miRNA is highly regulated at multiple levels including transcriptional and posttranscriptional regulation. A variety of posttranscriptional regulation occurs in biogenesis machinery of miRNA, which affect, the components of the miRNA processing machinery (Drosha, Dicer, argonote 2 or DGCR8), as example, impaired miRNA biogenesis enhance cellular transformation and promote tumorigenesis (6). Recent research has highlighted the role of genetic variation in miRNA genes and components of the biogenesis machinery in alteration of miRNA processing, which ultimately affects the levels of mature TS miRNAs in cancer. Deregulation of the expression of miRNAs can occur from aberrant transcription factor activity in cancer cells. Numerous Pol II–associated transcription factors are involved in the transcriptional control of miRNA genes. Some miRNAs are therefore under the control of tumor–suppressive or oncogenic transcription factors. Intriguingly, miRNAs have been shown in many studies to have a role in many transcription factor networks in cancer, representing a complex feedforward loop.

The activity of TS miRNA is not confined to the cancer cell but is extended to TME. In addition to these mechanism, cancer cells deregulate the expression of some miRNA in TME which creates an immunosuppressive microenvironment. TS miRNA have been shown to act directly by affecting cancer cell growth and viability but also indirectly by affecting angiogenesis and metastasis *via* secretion into the TME. These miRNAs regulate different stromal cells and exert their impact to recruit or promote the differentiation of suppressive immune cells, such Treg, CAF, TAMs and others that are involved in the TME (123). Reduced O_2_ availability (hypoxia) in the TME was shown to exert major regulatory effect in the expression of TS miRNA in TME.

As has been shown through this review, TS miRNAs have an important role as biomarkers in diagnosis and prognosis and have prominent clinical implications. Nevertheless, studies of miRNA-mediated interactions between cancer cells and stromal cells, specifically those focused on understanding the origin of miRNA alterations, are needed to improve targeted therapy.

## Author Contributions

KO, first author writer of the review. PL, supervision and correction of the work. All authors contributed to the article and approved the submitted version.

## Conflict of Interest

The authors declare that the research was conducted in the absence of any commercial or financial relationships that could be construed as a potential conflict of interest.

## Publisher’s Note

All claims expressed in this article are solely those of the authors and do not necessarily represent those of their affiliated organizations, or those of the publisher, the editors and the reviewers. Any product that may be evaluated in this article, or claim that may be made by its manufacturer, is not guaranteed or endorsed by the publisher.
